# Impact of Ground-Applied Termiticides on the Above-Ground Foraging Behavior of the Formosan Subterranean Termite

**DOI:** 10.3390/insects7030043

**Published:** 2016-08-26

**Authors:** Gregg Henderson, Bal K. Gautam, Cai Wang

**Affiliations:** Department of Entomology, Louisiana State University Agricultural Center, 404 Life Sciences Building, Baton Rouge, LA 70803, USA; bkgautam@uga.edu (B.K.G.); wangcai@scau.edu.cn (C.W.)

**Keywords:** aerial nest, *Coptotermes formosanus*, top foraging site, bottom foraging site, termiticide

## Abstract

We conducted a laboratory study to determine the impact of ground-applied termiticides on the above-ground foraging behavior of *Coptotermes formosanus*. Two concentrations (1 and 10 ppm) each of three termiticides, viz. fipronil, imidacloprid and chlorantraniliprole, were tested. After one month post-treatment (fipronil 10 ppm was run for 12 days only and all other treatments were run for one month), fipronil had the lowest percentage of survival (3%–4%) at both concentrations. Termite survival ranged from 31% to 40% in the case of imidacloprid treatments and 10 ppm chlorantraniliprole. However, 1 ppm chlorantraniliprole did not cause significant mortality compared to the controls. Foraging on the bottom substrate was evident in all replicates for all chemicals initially. However, a portion of the foraging population avoided the ground treatment toxicants after several days of bottom foraging. Only the slower-acting non-repellents created this repellent barrier, causing avoidance behavior that was most likely due to dead termites and fungus buildup on the treated bottom substrate. Fipronil appeared more toxic and faster acting at the concentrations tested, thus limiting this repellent effect. Suggestions by the pest control industry in Louisiana that some non-repellents can create a repellent barrier stranding live termites above ground are supported by this laboratory study.

## 1. Introduction

Soil treatments with non-repellent slow-acting liquid termiticides have been the dominant method of subterranean termite treatment [1]. Although termite baiting systems are typically aimed to suppress or eliminate termite colony populations from an area, the success of termite baits is often unpredictable as termites may not find and forage on the baits but may still find their way into the structure needing protection. Our earlier study [2] showed that a spot treatment of soil with a liquid and dust formulation of fipronil effectively killed termites that were not in the treatment site, suggesting that this termiticide is transferred among the members of the foraging population.

Although subterranean termites live underground by nature, construction of above-ground nests is not uncommon for *Coptotermes formosanus* Shiraki (Isoptera: Rhinotermitidae), but it is for native subterranean termites [3]. Typically Formosan subterranean termites will move from the soil carton nest to above-ground nests in a home or a tree, maintaining satellite nests connected via shelter tubes both above and below ground. True aerial nests, defined as an above-ground nest that has no connection to the ground [4], are less common. Aerial nests are most commonly the result of a connection break from the ground colony caused by a disturbance. Aerial colonies can also begin from the winged king and queen starting an aerial nest without ever having been in contact with the ground, or by worker termites bringing the queen and king to an above-ground site before breaking ground contact [4,5]. However, determining that the colony never had ground contact is not usually possible and only anecdotal estimates of such events are provided in the literature.

Control of termites in above-ground nests is very challenging, as it is difficult to find them for treatment. The options that became available in the mid-1990s were to treat the soil with either bait, or a slow-acting non-repellent termiticide with the likelihood of toxicant transfer through trophallaxis, mutual grooming or both. Previous studies focused on this aspect have had mixed results. Some studies [6,7,8,9,10] have reported success in suppressing termite populations well beyond the treated areas, while some [11,12,13] cast doubt on the long-distance impact of these termiticides. Despite differences in interpretation, it is established that slow-acting non-repellent termiticides have an impact beyond the treated site. No documented information, however, is available that *C. formosanus* in above-ground nests will not be impacted by a ground soil treatment with a non-repellent termiticide. The aim of this study is to determine the effects of ground-applied termiticides (fipronil, imidacloprid and chlorantraniliprole) on the above-ground foraging behavior of *C. formosanus* in artificial arenas in the laboratory.

## 2. Materials and Methods

Commercial formulations of three termiticides, fipronil (Termidor^®^ SC, 9.1% active ingredient (a.i.), BASF Corp., Ludwigshafen, Germany), imidacloprid (Premise^®^ 75 WP, 75% a.i., Bayer, Leverkusen, Germany) and chlorantraniliprole (Altriset™, 18.4% a.i., previously DuPont and now Syngenta, Basel, Switzerland) were tested in this study. These termiticides were already available in the lab and had been used for other experiments. Foraging groups of *Coptotermes formosanus* Shiraki were collected in October 2014, from Brechtel Park, New Orleans, LA, USA using milk crate traps [14]. In short, milk crates loaded with wood sticks arranged in lattice structure were placed in a known termite infested area and checked after two months. The infested crates were retrieved and brought to the lab and held in trash cans. Water was added when necessary to maintain the wood moisture and high relative humidity inside the cans. One to two days prior to the bioassays, termites were dislodged from the wood and collected in a clean container lined with moist paper towels. Termites collected from a single colony were used for this study.

**Figure 1:**
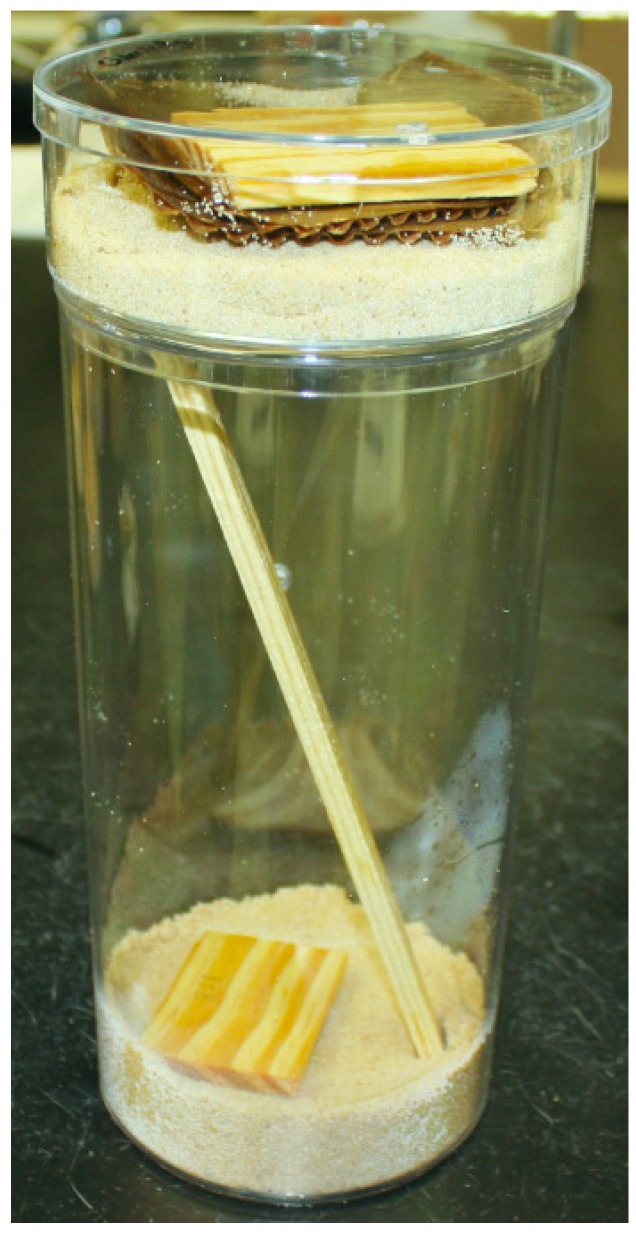
Bioassay arena showing top and bottom foraging sites connected with a ramp.

The bioassay arena consisted of two foraging sites, a bottom site and top site, connected by a 21 cm wooden ramp so that termites could readily move from one site to another (Figure 1). To prepare the top foraging site, a round clear acrylic container (11 cm diameter × 4 cm high, Pioneer Plastics^©^, North Dixon, KY, USA) with a hole on bottom one side was lined with a single layer of filter paper. The container was partly filled with autoclaved sand (~400 g) and distilled water was added to make the moisture content ~10%. A block of *Pinus* wood (3 cm × 4 cm× 1 cm) followed by a disc of corrugated cardboard, both autoclaved and weighed, were placed on sand surface. The total amount of food materials provided (top wood, top cardboard, ramp and bottom wood) was identical (≈30 g) for all the bioassay arenas. The cardboard was moistened by adding 2 mL of distilled water. A small hole was made on the lid of the top container to facilitate in adding water whenever deemed necessary.

To prepare the bottom foraging site, a tall round clear acrylic container (11 cm diameter × 21 cm high, Pioneer Plastics©, North Dixon, KY, USA) was partly filled with ~400 g of autoclaved treated sand and a wood block of similar size as on the top container was placed on the sand surface. Three chemicals each with two concentrations, 1 ppm and 10 ppm were tested. To treat the sand, first autoclaved sand was held in a sealable plastic bag and the required amount of chemical mixed with distilled water (the amount of distilled water was calculated to make the moisture content of sand 10%) was added. The bag was then sealed and kneaded thoroughly to mix the chemicals uniformly in the substrate. Controls received moist sand with no added termiticide. A small hole was made about the middle height on one side of the container to add water when necessary. A slit was made on the lid in a way that it was aligned with the hole of the top container. A 21-cm-long wooden stake was used as a ramp connecting the top and the bottom foraging sites. One end of the stake was placed on the surface of the bottom substrate and the other end was inserted through the hole to reach the substrate on the top container so that the ramp connects at an angle of ~60°.

Fourteen hundred termites (in their collected worker/soldier/nymph ratio) were introduced on the top container and the lid placed back on. Three replications were prepared for each treatment and control, for a total of 21 bioassay arenas and ~30,000 termites in this study. The test arenas were placed undisturbed in the lab at 23 ± 2 °C. All the bioassays ran for one month, unless mortality appeared to be 100% prior to that date.

Termite movement between the top and the bottom foraging sites was recorded. For this, the number of termites making upward and downward movement in a 5 min period was recorded every day from day 2 to day 7 and then at day 20 and day 30. The bottom side of the bottom foraging container was scanned at day 2, day 7 and day 30 to measure progress in tunneling over time if any was present. During the test period, notable observations like aggregation of termites and fungus growth on dead termites were recorded. After one month, we measured the length of tunnels (using a scanner) in the lower end of the bottom foraging site, dismantled arenas and counted live termites in all sections of the arenas. Wood and cardboard were cleaned and dried to calculate the consumption.

Data analysis was done using Proc GLM in SAS 9.3. (SAS Institute Inc., Cary, NC, USA) Mortality data were arcsine of the square root transformed to improve normality for the analysis. Termite movement observed for 5 min period on the ramp each day was analyzed separately and compared among the treatments for the same day. Post-ANOVA means comparison was done using Tukey’s honestly significant difference at α = 0.05.

## 3. Results

### 3.1. Termite Movement

**Figure 2:**
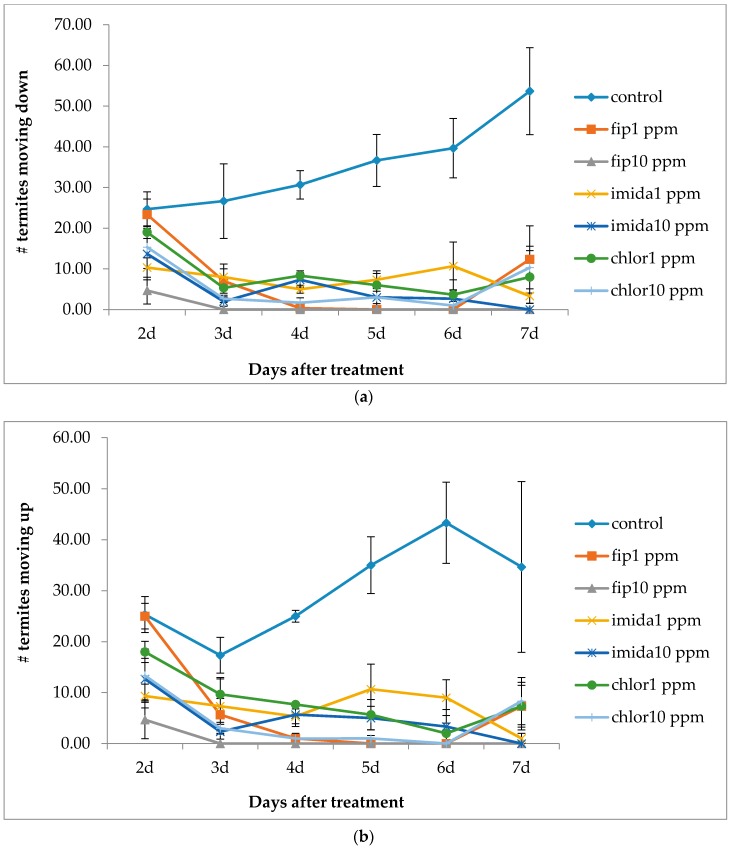
(**a**) Mean number of termites (±SEM) moving downward on the ramp in 5 min observation period from day 2 to day 7 after treatment; (**b**) Mean number of termites (±SEM) moving up on the ramp in 5 min observation period from day 2 to day 7 after treatment.

**Figure 3:**
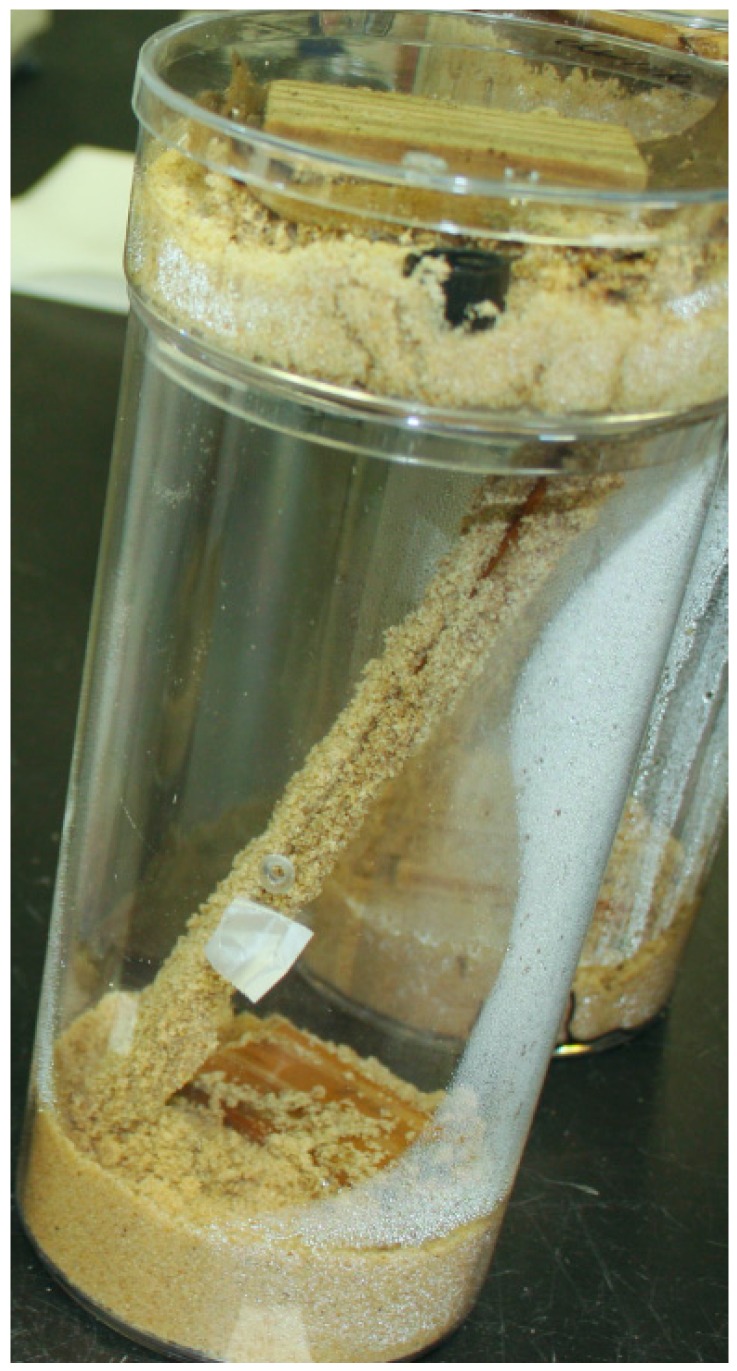
Shelter tube construction and termites on ramp at chlorantraniliprole 1 ppm treatment.

Within 1 h of the release into the top container, all the treatments had termites foraging in the bottom containers and moving up and down along the connected wooden ramps. When compared with the controls, the number of termites moving up or down the ramp was significantly lower in all termiticide treatments by day 4 and thereafter (upward movement: df = 6, 14; *F* = 19.52; *p* < 0.0001; downward movement: df = 6, 14; *F* = 27.11; *p* < 0.0001) until day 7 (Figure 2). At day 8 and after, termites in the controls and chlorantraniliprole 1 ppm treatments built shelter tubes over the full length of the ramps (Figure 3), so recording of up and down movement was not possible for these groups at day 8. For the rest of the treatments, the number of termites moving up and down remained very low after one week. At day 20, one replication of imidacloprid 10 ppm had a full-length shelter tube on the ramp, and at day 30, one replication of imidacloprid 1 ppm also had a full-length shelter tube. All of the shelter tubes, once built on the ramps, remained intact until the end of the experiment.

### 3.2. Termite Mortality

**Figure 4:**
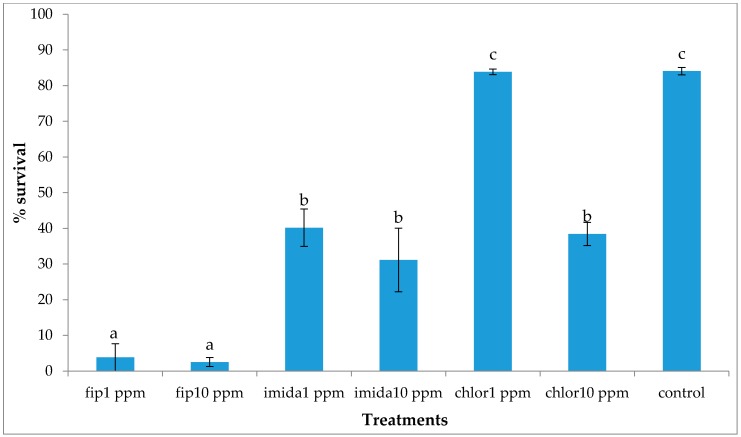
Mean % survival (±SEM) at one month (12 days for fip10 ppm) after treatment. Means with the same letters are not significantly different (*p* > 0.05) using Tukey’s HSD means separation.

Mortality was first observed at day 2 in the 10 ppm fipronil treatments and at day 3 in the 1 ppm fipronil treatments. Although accurate counting was not possible, some dead and dying termites were observed, especially on the bottom foraging substrate. All other treatments had no dead termites observed until day 5. Interestingly, the majority of the termites were aggregated on the bottom substrate in the 10 ppm chlorantraniliprole and 10 ppm imidacloprid treatments. After one week, dead termites were covered with fungus on both the bottom and the top foraging sites of the fipronil treatments. Similarly, the bottom site of the imidacloprid treatments and 10 ppm chlorantraniliprole also had fungus growth. For the 10 ppm fipronil treatments, since no signs of live termites were noticed after one week, all three replications were taken down at day 12. Surprisingly, when the arenas were dismantled, some live termites (range: 15–70) were retrieved from the top foraging site of all the replications. Survival count at the end of the experiment (one month) showed that both concentrations of fipronil treatments had the lowest survival (3%–4% survival) followed by both concentrations of imidacloprid and the 10 ppm chlorantraniliprole treatments, all being significantly lower compared to controls or chlorantraniliprole 1 ppm (df = 6, 14; *F* = 57.33; *p* < 0.0001). Termite survival in 1 ppm chlorantraniliprole (83%) was not significantly different than that in the controls (Figure 4).

### 3.3. Location of Live Termites

**Figure 5:**
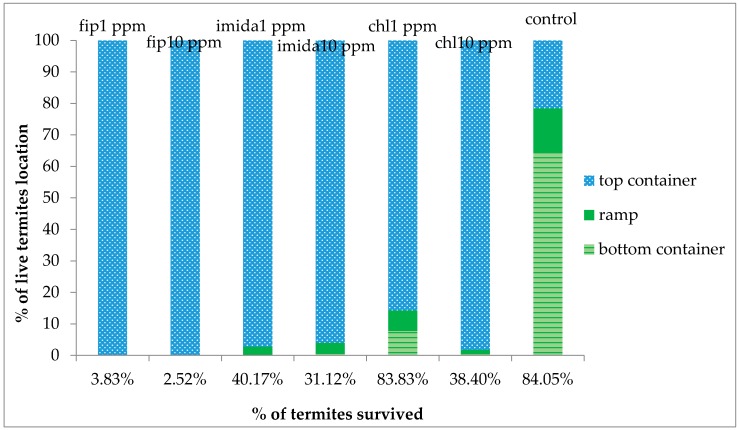
Percentage of live termites located among the three sites (top foraging substrate, ramp and bottom foraging substrate) after one month (12 days for fip10 ppm) of treatment. Y-axis is the total percentage of live termites retrieved after one month of treatment.

At the end of the bioassay period, only control arenas had termites more or less equally distributed between the top and the bottom containers. Among the termiticidal treatments, the 1 ppm chlorantraniliprole treatment had a small percentage (8%) of live termites retrieved at the bottom substrate, whereas all other treatments had termites solely present at the top substrate (Figure 5).

### 3.4. Food Consumption

**Figure 6:**
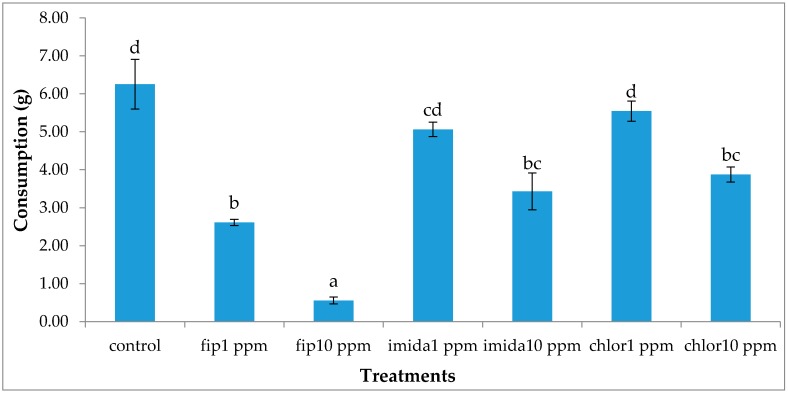
Mean total food (top wood, top cardboard, ramp and bottom wood) consumption (±SEM) after one month (12 days for fip10 ppm) of treatment. Means with the same letters are not significantly different (*p* > 0.05) using Tukey’s HSD means separation.

**Figure 7:**
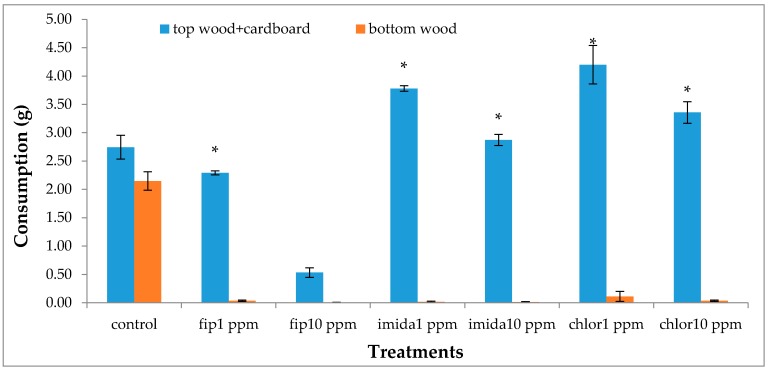
Comparison of mean food consumption (±SEM) between top foraging site and bottom foraging site within the same treatment after one month (12 days for fip10 ppm) of treatment. Top foraging site consumption included consumption of wood and cardboard and bottom foraging site consumption included consumption of wood only as there was no cardboard there. Asterisk (*) on top of graph bars shows significant difference between top and bottom foraging sites within the same treatment.

Termiticidal treatments had a significant impact on the total consumption (df = 6, 14; *F* = 31.85; *p* < 0.0001). In general, the total consumption corresponded to the number of live termites retrieved at the end of the experiment. The highest total consumption was found in the controls which was significantly higher than fipronil treatments or 10 ppm imidacloprid, but the difference was not significant with the 1 ppm imidacloprid or chlorantraniliprole treatments (Figure 6). The location (top or bottom container) of food also had a significant impact on consumption (df = 13, 28; *F* = 146.18; *p* < 0.0001). Consumption was not significantly different between the top and bottom containers in the controls, whereas consumption was almost entirely on the top foraging site in the rest of the treatment sets, except 10 ppm fipronil, which had no measurable consumption (Figure 7).

### 3.5. Tunnel Construction

**Figure 8:**
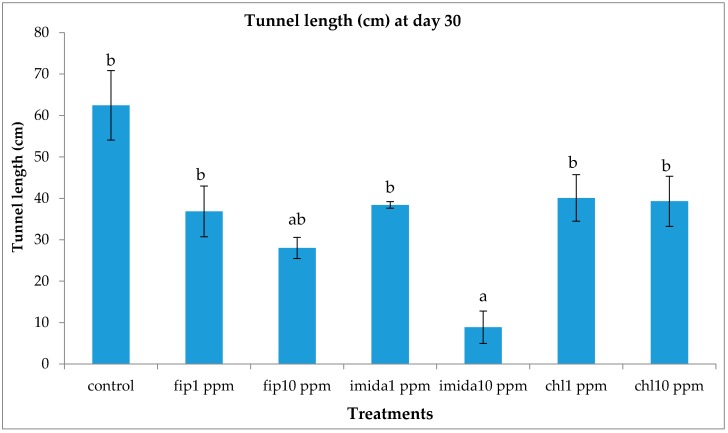
Mean tunnel length (±SEM) on the bottom foraging container at the end of the one-month (12 days for fip10 ppm) test. Means with the same letters are not significantly different (*p* > 0.05) using Tukey’s HSD means separation.

Treatments had a significant effect on the length of the tunnels on the bottom foraging container measured at the end of the experiment (df = 13, 28; *F* = 146.18; *p* < 0.0001). The tunnel length was significantly shorter in the 10 ppm imidacloprid treatment compared to the five other treatments and controls. The five treatments had more or less similar tunnel lengths, which while shorter than the controls, the difference was not statistically significant (Figure 8).

## 4. Discussion

In the present study we found that all colony units attempted to make ground contact despite the treatments that were in place. Most treatments caused repellency prior to colony death and only fipronil was able to induce a high mortality to an above-ground colony making ground contact.

The delayed action property of termiticides is usually considered an advantage over fast action as it allows termites to live longer, thereby allowing more time to interact with other nestmates in the colony. However, based on our results, the comparatively faster action of fipronil (comparison made within the same concentration) may be desirable in some situations as termites would be killed before they get a chance to avoid the piles of dead nestmates, usually covered with fungus. Even with the relatively fast action of fipronil, a small proportion of termites were alive at the end of the experiment and looked healthy and active. These live termites were living at the bottom of the top substrate, apparently avoiding the area where dead termites were present and fungus growth was observed, suggesting that *C. formosanus* manages to avoid the mortality-causing factors. It was likely that the termites that were not dead at the end of the one-month tests would have survived normally had we extended the test period.

The lower concentration of the chlorantraniliprole (1 ppm) treatment did not cause significant mortality in termites and termite activities, such as shelter tube construction on ramps, were unaffected. Previous studies have reported that chlorantraniliprole has a more delayed action compared to fipronil or imidacloprid [15,16]. At 10 ppm, however, chlorantraniliprole had a similar mortality as imidacloprid. Buczkowski et al. [17] reported that 5 ppm chlorantraniliprole treatments caused significant mortality in donors but not in the recipients, suggesting that the chemical has a dose-dependent toxicity and delayed action.

For all three chemicals tested, a proportion of the released termites were alive at the end of the experiment and they looked healthy and active. The live termites were all on the top foraging sites, except a small proportion that was retrieved from the upper part of the ramps. None were collected from the bottom foraging sites which had the treated substrate. We suspect that different impacts may be seen between termites traveling a vertical distance versus a horizontal distance. Previous researchers [10,12,13] conducted laboratory studies to test the mortality of termites beyond the treated site based on horizontal distance alone. There are no reports of using vertical distance measures. Su [12] reported the clogging/blockage of tunnels by dead termites, thus severing the contact after a certain percentage of termites were dead. There was no clogging/blockage in our study as no shelter tubes were constructed on ramps in the treatments except in the 1 ppm chlorantraniliprole treatment. However, the number of termites moving down the ramp was fewer and fewer as the days passed by and almost no termite movement was observed at or after 20 days of treatment. We suggest that the cessation of termite movement to the bottom container was due to the repellent effects from dead termites covered with fungus.

Imidacloprid treatments seemed to show an immediate effect on termite behavior, especially at 10 ppm, as the tunneling on the bottom foraging substrate was significantly lower compared to other termiticide treatments. Interestingly, while termite mortality at this treatment was slower compared to the fipronil treatments (1 or 10 ppm), they almost immediately aggregated and stopped activities such as movement or tunnel construction. Although such an aggregation on the bottom container was observed in the 10 ppm chlorantraniliprole treatments also, some termites were observed digging into the substrate, apparently engaging in tunnel construction. The finding that 10 ppm imidacloprid treatment impacted termite activity quickly is consistent with the finding by the author of [18] who reported that imidacloprid treatments bring about an immediate change in *C. formosanus* behavior.

## 5. Conclusions

The aim of a ground treatment with non-repellent compounds such as fipronil, imidacloprid and chloratraniliprole is not only to create a toxic barrier to keep healthy termites from entering the house, but also to impact the termites that are already within the house that have formed above-ground satellite nests. The results from this study showed that the impact of ground-applied termiticides on an above-ground *C. formosanus* population is dependent on the type and concentration of the termiticides. Fipronil caused the highest mortality of termites released on the top foraging chamber as the majority of the termites were killed before they were repelled by the dead nestmates, followed by imidacloprid. Chlorantraniliprole, especially at the lower concentration, did not cause significant mortality as compared to the controls. This is probably due to the relatively slow action of chlorantraniliprole that created a repellent barrier as a result of dead termites covered with fungus causing avoidance behavior. Fipronil appeared more toxic and faster acting at the concentrations tested, thus limiting this repellent effect and impacting the maximum number of nestmates. Suggestions by the pest control industry in Louisiana that some non-repellent termiticides can create a repellent barrier stranding live termites above ground are supported by this laboratory study.
